# A Meta-analysis Comparing Toothbrush Technologies on Gingivitis and Plaque

**DOI:** 10.1016/j.identj.2023.06.009

**Published:** 2023-07-21

**Authors:** Yuanshu Zou, Julie Grender, Ralf Adam, Liran Levin

**Affiliations:** a Oral Care Research & Development, The Procter & Gamble Company, Mason, Ohio, USA; b Oral Care Research & Development, Procter & Gamble Service GmbH, Kronberg, Germany; c Faculty of Medicine and Dentistry, University of Alberta, Edmonton, Alberta, Canada

**Keywords:** Biofilm, Bacteria, Prevention and control, Toothbrushing, Gingival disease, periodontitis

## Abstract

**Background:**

Research continues to show an association between oral health and systemic health, further stressing the importance of effective daily plaque removal via toothbrushing to maintain periodontal health and overall well-being. This investigation was undertaken to compare the efficacy of oscillating-rotating, sonic, and manual toothbrushes in reducing gingivitis and plaque in randomised controlled trials (RCTs) with up to 6 months’ follow-up.

**Methods:**

This meta-analysis was conducted from a single database (Procter & Gamble Oral Care Clinical Archive) including RCTs from 2007 to 2022. Three authors independently assessed study eligibility. Disagreements concerning selected studies were resolved by discussion with an expert colleague. Direct and indirect treatment comparisons along with transition rates to gingival health were calculated using participant-level data. Transition-to-health time was calculated using data from all time points. Subregion analyses evaluated number of bleeding sites and plaque reduction.

**Results:**

This meta-analysis included 21 gingivitis RCTs and 25 plaque RCTs. Relative to manual and sonic brushes, oscillating-rotating brushes had a higher percentage of participants who transitioned to gingival health (72% vs 21% and 54%; *P* < .001). Compared with manual and sonic brushes, respectively, oscillating-rotating brushes demonstrated greater bleeding site reductions (by 52% and 29%; *P* < .001) and superior plaque reductions (by 19% and 5%; *P* < .001). Oscillating-rotating brushes provided faster transitions to health than sonic brushes and showed greater efficacy across subregions. The most advanced oscillating-rotating brush demonstrated statistically significantly greater efficacy compared with traditional oscillating-rotating, manual, and sonic brushes when analysed separately. Risk of bias was deemed low for all studies.

**Conclusions:**

Oscillating-rotating toothbrushes offer superior results for transition to health, gingivitis, and plaque reduction compared with manual and sonic brushes. The most advanced oscillating-rotating model offers enhanced efficacy vs traditional models.

## Introduction

Bacterial plaque triggers gingivitis, an oral inflammatory response marking the onset of periodontal disease,^1^ which is a widespread global issue.^2^ Effective toothbrushing removes dental plaque^3^ to prevent and reverse gingivitis, thereby reducing the risk of periodontitis, which has been associated with systemic conditions such as cardiovascular disease, diabetes, and Alzheimer's disease.^4^ Therefore, for the individual, removing plaque and preventing gingivitis have the potential to improve not only oral health but also whole-body health. For society, a cost-benefit analysis^5^ established that the economic burden of periodontitis is mitigated better by a strategy of prevention than of treatment. Prevention also ameliorates the environmental burden of dentistry, contributing towards an ecologically sustainable dental practice over time.^6^

Twice-daily brushing with a fluoride toothpaste is accepted as the basic standard of care for at-home plaque removal. Whilst a well-designed manual toothbrush can provide thorough plaque removal, the effectiveness of this device is highly sensitive to users’ brushing behaviour and compliance with professional guidance.^7^ Over time, a variety of toothbrush designs have been developed to optimise plaque control, including electric toothbrushes. Electric brushes have demonstrated consistently greater gingivitis reduction and plaque removal compared with manual brushes in systematic reviews.^3^^,^^8^, ^9^, ^10^ An 11-year observational study also found that electric toothbrush users exhibited reduced clinical attachment loss progression and periodontal pocket depth as well as greater tooth retention compared with manual brush users.^11^

Electric toothbrushes have evolved to include built-in timers, pressure sensors, expanded brushing modes, and Bluetooth-connected apps to increase brushing time and thoroughness.^12^^,^^13^ The majority of contemporary electric toothbrushes are classified as sonic (side-to-side motion) or oscillating-rotating (O-R). Evidence shows greater gingivitis and plaque reduction for the latter technology.^8^^,^^14^, ^15^, ^16^, ^17^ A prior meta-analysis assessed the gingivitis- and plaque-reducing effects of O-R vs manual and sonic electric toothbrushes.^15^ That analysis focused on 20 randomised controlled trials (RCTs) evaluating plaque and 16 RCTs assessing gingivitis, all of which were conducted between 2007 and 2017. Access to participant-level data was required from every study to assess the relationship between baseline and end-of-treatment gingivitis levels for each tested toothbrush. In 2020, an O-R brush model (iO; Oral-B iO, Procter & Gamble) was introduced with a linear magnetic drive replacing the traditional O-R gear-based mechanical drive system. The linear magnetic drive produces micro-vibrations at bristle tips, which might augment plaque removal.^12^

In light of the significant design changes associated with the latest O-R model, this investigation was undertaken to extend the reach of the prior meta-analysis and compare the efficacy of O-R toothbrushes, including the latest model, with manual negative control and sonic positive control toothbrushes in reducing gingivitis and plaque in RCTs with up to 6 months’ follow-up. It also offers new perspectives on the data. Whilst the previous analysis characterised gingivitis solely by number of bleeding sites, the new analysis additionally reflects changes in patients’ modified gingival index (MGI) scores. New by-region analyses of number of bleeding sites and Rustogi Modified Navy Plaque scores shed light on brush efficacy in hard-to-reach areas. Additionally, the current study analyses participant-level data from all time points in each study (vs only baseline and final time points) to assess how quickly patients transitioned to a gingival health state. Finally, this new meta-analysis includes toothbrushing efficacy evaluations over a longer usage period (up to 6 months), whereas the prior meta-analysis included studies up to 3 months.

## Materials and methods

This study was conducted in accordance with the PRISMA statement^18^ and is registered on ClinicalTrials.gov (NCT05594004). All RCTs included in this analysis were conducted according to protocols approved by Ethics Committees and in compliance with the International Conference on Harmonization (ICH) and Good Clinical Practice (GCP) principles.

### Search process

The authors searched the Procter & Gamble Oral Care Clinical Archive for eligible RCTs from 2007 to 2022, which includes new studies and those in the prior meta-analysis.^15^ Trials in the archive were reviewed to determine their eligibility for inclusion in the meta-analysis. This meta-analysis was not associated with a systematic review because individual participant-level data were required.

### Eligibility criteria

Studies that met the following criteria were eligible: (1) up to 6 months’ duration; (2) randomised and controlled, parallel-group, and examiner-blinded design; (3) reported plaque and/or gingivitis outcomes after an intervention with comparator control group(s); and (4) examiner-based. Digital imaging studies were excluded. All individual studies excluded participants with severe periodontal disease, as characterised by purulent exudate, generalised mobility, and/or severe recession.

### Study selection and data collection

Three authors (RA, JG, YZ) independently assessed study eligibility. Disagreements concerning selected studies were resolved by discussion with an expert colleague. From selected studies, the following data were collected for both intervention and control groups: study name, year, country, and design; age and sex of each participant; experimental and comparator treatments; timing of follow-up visits; and outcome measurements (participant-level data).

### Risk of bias assessment

The quality of each RCT was assessed using the Cochrane risk-of-bias tool (RoB2) for randomised parallel-group trials.^19^

### Statistical analysis

Gingivitis data were expressed in terms of number of bleeding sites and MGI score.^20^ As in the previous meta-analysis,^15^ the number of bleeding sites was calculated using the Löe-Silness Gingival Index, Gingival Bleeding Index, Papillary Bleeding Index, or Mazza Gingival Index within each study. If there was more than 1 follow‐up visit in the trial, the final assessment up to and including the 6-month visit was used for data extraction for all analyses except the time-to-transition-to-health analysis, which included all assessments. One-step, participant-level meta-analysis used a mixed model for direct and indirect treatment comparisons and for identification of participant-level covariates.^21^ Study and treatment were included as random effects, allowing for different treatment-effect sizes by study. Baseline gingivitis score and separate interactions with study and treatment were also modelled to allow the relationship between baseline and end-of-treatment gingivitis score to differ by study and treatment. The 1-step model allowed for between-study variability of the residual variance (Equation 9).^22^ Adjusted treatment mean gingivitis scores with standard error bars and estimated mean difference between treatments with *P* values and 95% confidence intervals (CIs) were included in the bar plots with tables. Percentage changes from control were calculated using adjusted mean scores. Similar analyses were also generated for lingual, buccal, molar, molar-lingual, and molar-buccal subregions.

Classification of gingival status as healthy (<10% bleeding sites), localised gingivitis (10%−30% bleeding sites), or generalised gingivitis (>30% bleeding sites) was based on gingivitis case definitions established in 2017.^1^ Rates of transition between classes were calculated for each treatment, and odds ratios were generated with 95% CIs. To assess time to reaching gingival health status, all interim time point bleeding data were used for the time-to-event analysis with event defined as transition to gingival health (<10% bleeding sites). The cumulative incidence of event (F(t)) is described as the probability that an event has occurred by time t, ie, F(t) = 1-S(t)= Pr(*T* ≤ *t*) with S(t) as the survival function. The cumulative incidence curve is therefore the complement of the survival curve which can be estimated from Kaplan–Meier estimator. Data are considered as censored when a participant does not experience a transition to health by the end of the study. The cumulative incidence curve is plotted with 95% CIs for each treatment and overall treatment comparison is generated using the Log-rank test.^23^

Per the prior meta-analysis,^15^ plaque data, expressed as Turesky Modification of the Quigley-Hein Index or the Rustogi Modified Navy Plaque Index, were standardised by dividing each study's mean treatment difference by the respective standard deviation.^24^ Direct and indirect comparisons of treatments were accomplished by network meta-analysis; the requisite assumptions of homogeneity, transitivity, and treatment rank credibility were confirmed as satisfied. Network meta-analysis produced treatment differences with *P* values, 95% CIs, and *P* scores based on point estimates and standard errors of the frequentist network meta-analysis estimates. *P* scores were calculated as averaged 1-sided *P* values and used to rank the treatments^25^; larger *P* scores indicate greater certainty that a given treatment has better antiplaque efficacy. Percentage change from control was calculated by the weighted percentage change from the control from each study with weights calculated from the random-effects model using within-study variance and between-study variance. A 1-step meta-analysis on Rustogi Modified Navy Plaque Index was also done for lingual, buccal, molar, molar-lingual, molar-buccal, interproximal, interproximal-anterior, interproximal-molar, and gingival subregions.

Summary-level and network meta-analyses used the “metafor” and “net Meta” packages in R version 3.2.3.^26^^,^^27^ Participant-level time to event analysis used “survival” and “surd miner” packages in the same R version. All other participant-level analyses were conducted using SAS 9.4 (SAS Institute).

## Results

### Clinical trials overview

This meta-analysis represents 21 RCTs from 3 countries, involving 2655 participants, wherein gingivitis was assessed. Eight trials compared O-R brushes with manual brushes; 13 trials compared O-R brushes with sonic brushes. There were 25 RCTs from 3 countries, involving 3019 participants, wherein plaque was assessed. Eleven trials compared O-R brushes with manual brushes; 14 trials compared O-R brushes with sonic brushes^28^, ^29^, ^30^, ^31^, ^32^, ^33^, ^34^, ^35^, ^36^, ^37^, ^38^, ^39^, ^40^, ^41^, ^42^, ^43^, ^44^, ^45^, ^46^, ^47^, ^48^, ^49^ (see Table 1).

**Table 1:** Randomised controlled trials included in the meta-analyses, listed in reverse chronological order.

Study	Location	Duration	Population, inclusion criteria	Outcomes measures	Control toothbrush
Grender et al^28^	Ontario, Canada	12 weeks	Adults *N* = 100 BS: 20–90 MGI: 1.75–2.5 RMNPI: >0.5	GBI, MGI, RMNPI	Manual: Oral-B Indicator
Grender et al^29^	Ontario, Canada	12 weeks	Adults *N* = 100 BS: 20–90 MGI: 1.75–2.5 RMNPI: >0.5	GBI, MGI, RMNPI	Manual: Oral-B Indicator
Goyal et al^30^	Ontario, Canada	6 months	Adults *N* = 110 BS: 20–90 MGI: 1.75–2.5 RMNPI: >0.5	GBI, MGI, RMNPI	Sonicare DiamondClean
Adam et al^31^	Ontario, Canada	8 weeks	Adults *N* = 90 BS: 20–90 MGI: 1.75–2.5 RMNPI: >0.5	GBI, MGI, RMNPI	Sonicare DiamondClean
Grender et al^32^	Ontario, Canada	8 weeks	Adults *N* = 110 BS: ≥20 MGI: ≥1.75 RMNPI: >0.5	GBI, MGI, RMNPI	Manual: ADA reference
Ccahuana-Vasquez et al^33^	Ontario, Canada	5 weeks	Adults *N* = 150 BS: ≥10 MGI: 1.75–2.3 RMNPI: ≥0.5	GBI, MGI, RMNPI	Manual: ADA reference
Ccahuana-Vasquez et al^34^	Ontario, Canada	8 weeks	Adults *N* = 148 BS: ≥10 MGI: 1.75–2.3 RMNPI: ≥0.5	GBI, MGI, RMNPI	Sonicare DiamondClean
Erbe et al^35^	Mainz, Germany	2 weeks	Adolescents *N* = 59 TMQHPI: ≥1.75	TMQHPI	Manual: Oral-B 35 Indicator
Adam et al^36^	Indiana, USA	8 weeks	Adults *N* = 95 TMQHPI	TMQHPI	Manual: ADA reference
Li et al^37^	Beijing, China	3 months	Adults *N* = 123 BS: ≥15	Mazza GI	Manual: Lion Dentor Systema
Ccahuana-Vasquez et al^38^	Ontario, Canada	8 weeks	Adults *N* = 148 BS: ≥10 MGI: 1.75–2.3 RMNPI: ≥0.5	GBI, MGI, RMNPI	Sonicare DiamondClean
Goyal 2015, unpublished	Ontario, Canada	4 weeks	Adults *N* = 97 RMNPI: ≥0.5	GBI, MGI, TMQHPI	Sonicare DiamondClean
Klukowska et al^39^	Indiana, USA	6 weeks	Adults *N* = 94 TMQHPI ≥1.75	TMQHPI	Manual: ADA reference
Klukowska et al^40^	Ontario, Canada	12 weeks	Adults *N* = 127 BS: ≥10 MGI: 1.75–2.3 RMNPI: ≥0.5	GBI, MGI, RMNPI	Sonicare FlexCare Platinum
Klukowska et al^41^	Nevada, USA	6 weeks	Adults *N* = 128 BS: ≥10 MGI: 1.75–2.3 RMNPI: ≥0.5	GBI, MGI, RMNPI	Colgate ProClinical A1500
Klukowska et al^42^	Ontario, Canada	6 weeks	Adults *N* = 128 BS: ≥10 MGI: 1.75–2.3 RMNPI: ≥0.5	GBI, MGI, RMNPI	Sonicare DiamondClean
Büchel et al^43^	Jena, Germany	4 weeks	Adults *N* = 129 TMQHPI: ≥1.75	TMQHPI	Colgate ProClinical C200
Milleman 2014, unpublished	Indiana, USA	4 weeks	Adults *N* = 97 BS: ≥20 TMQHPI: ≥1.8	GBI, MGI, TMQHPI	Manual: ADA reference
Klukowska et al^44^	Ontario, Canada	4 weeks	Adults *N* = 99 BS: ≥10 MGI: 1.75–2.3 RMNPI: ≥0.5	GBI, MGI, RMNPI	Manual: ADA reference
Klukowska et al^45^	Nevada, USA	12 weeks	Adults *N* = 127 BS: ≥10 MGI: 1.75–2.3 RMNPI: ≥0.5	GBI, MGI, RMNPI	Colgate ProClinicalA1500
Sigusch 2013, unpublished	Jena, Germany	6 weeks	Adults *N* = 99 TMQHPI: ≥1.75	TMQHPI	Manual: ADA reference
Klukowska et al^46^	Ontario, Canada	12 weeks	Adults *N* = 130 BS: ≥10 MGI: 1.75–2.3 RMNPI: ≥0.5	GBI, MGI, RMNPI	Sonicare DiamondClean
Klukowska et al^47^	Ontario, Canada	4 weeks	Adults *N* = 117 MGI: 1.75–2.3 RMNPI: ≥0.5	GBI, MGI, RMNPI	Manual: ADA reference
Williams et al^48^	Missouri, USA	10 weeks	Adults *N* = 165 for LSGI; *N* = 176 for TMQHPI BS: ≥20 LSGI: ≥1.1 TMQHPI: ≥1.75	LSGI; TMQHPI	Sonicare FlexCare
Goyal et al^49^	Ontario, Canada	12 weeks	Adults *N* = 173 MGI: 1.73–2.3	GBI, MGI, RMNPI	Sonicare FlexCare
Putt 2007, unpublished	Indiana, USA	12 weeks	Adults *N* = 188 PBI: ≥1.1 TMQHPI: ≥1.75	PBI, TMQHPI	Sonicare FlexCare

GBI: Gingival Bleeding Index; LSGI: Löe-Silness Gingival Index; Mazza GI: Mazza Gingival Index; MGI: Modified Gingival Index; PBI: Papillary Bleeding Index; RMNPI: Rustogi Modified Navy Plaque Index; TMQHPI: Turesky Modification of Quigley-Hein Plaque Index.

#### Gingival bleeding sites

**Fig. 1:**
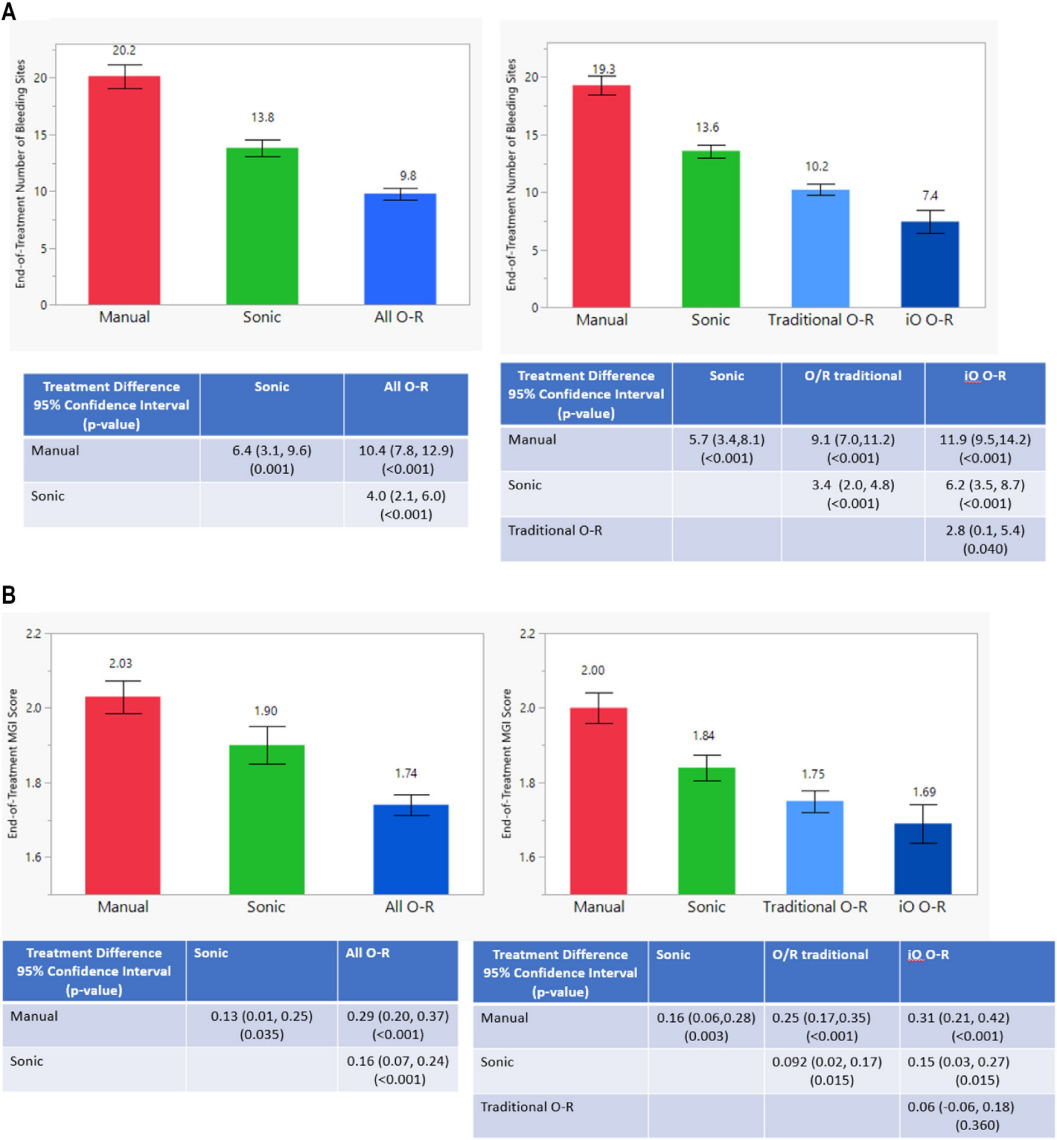
**A**, Adjusted mean end-of-treatment number of bleeding sites (standard error), treatment differences, confidence intervals, and *P* values using 1-step meta-analysis on subject-level data. **B**, Adjusted mean end-of-treatment modified gingival index (MGI) scores (standard error), treatment differences, confidence intervals, and *P* values using 1-step meta-analysis on subject-level data.

Figure 1A shows adjusted mean number of gingival bleeding sites at end of treatment per brush group. In studies assessing the effects of all O-R brushes (ie, iO O-R studies and traditional O-R studies combined) on gingivitis vs manual and sonic brushes, participants using an O-R brush had an average of 10.4 (95% CI, 7.8–12.9) fewer bleeding sites compared with a manual brush and 4.0 (95% CI, 2.1–6.0) fewer bleeding sites vs a sonic brush (*P* < .001) at end of treatment. These reductions equate to a 52% and 29% bleeding reduction benefit for O-R technology vs the respective controls. When the iO O-R brush subgroup was analysed separately, it demonstrated the fewest bleeding sites, followed by traditional O-R, sonic, and manual brushes, in that order (*P* ≤ .04). Study participants using an iO O-R brush had a bleeding reduction benefit of 62% vs manual, 46% vs sonic, and 27% vs traditional O-R. The use of a sonic brush yielded a reduction in average number of bleeding sites of 6.4 vs manual (*P* = .001) and a 32% bleeding reduction.

Baseline bleeding-by-treatment interaction term was significant (*P* < .001) in the subject-level 1-step meta-analysis model, indicating that the effect of an O-R electric brush compared with that of the control depends on the number of baseline bleeding sites. The specific nature of this relationship is summarised in Supplementary Figure 1. The figure shows that the relative efficacy across brushes remains consistent, with iO O-R being the most effective and manual being the least effective, regardless of baseline bleeding level. The figure also demonstrates that the benefit of an O-R brush increases as baseline bleeding increases.

In the transition-to-health analysis, 72% of all O-R brush users with gingivitis at baseline transitioned to healthy gingival status at end of treatment compared with 54% of sonic brush users and 21% of manual brush users (*P* < .001). Participants with baseline gingivitis had 9.5 times better odds (95% CI, 7.0–13.0) of transitioning to gingival health status using O-R compared with a manual toothbrush and 2.2 times better odds (95% CI, 1.7–2.7) vs a sonic toothbrush. When subgroups of O-R users were analysed separately, 88% of participants using iO O-R and 65% of participants using traditional O-R transitioned from baseline gingivitis to a state of gingival health (*P* < .001; Table 2). A more detailed analysis showing transitions across generalised gingivitis, localised gingivitis, and gingival health is shown in Table 3.

**Table 2:** Changes from baseline: baseline bleeding percentage vs postbaseline bleeding percentage.

			Postbaseline	
	Baseline	Total (n)	Gingival health	Gingivitis
**All O-R**	Gingival health	487	479 (98%)	8 (2%)
	Gingivitis	842	606 (72%)	236 (28%)
***O-R subgroups: iO O-R***	Gingival health	0	0 (0%)	0 (0%)
	Gingivitis	255	225 (88%)	30 (12%)
***Traditional O-R***	Gingival health	487	479 (98%)	8 (2%)
	Gingivitis	587	381 (65%)	206 (35%)
**Manual**	Gingival health	132	132 (100%)	0 (0%)
	Gingivitis	315	67 (21%)	248 (79%)
**Sonic**	Gingival health	344	321 (93%)	23 (7%)
	Gingivitis	535	290 (54%)	245 (46%)

Data shown use 2 classifications: gingival health (<10% bleeding sites) and gingivitis (≥10% bleeding sites).

O-R, oscillating-rotating.

**Table 3:** Changes from baseline: baseline bleeding percentage vs postbaseline bleeding percentage.

		Postbaseline			
	Baseline	Total (n)	Gingival health	Localised gingivitis	Generalised gingivitis
**All O-R**	Gingival health	487	479 (98%)	8 (2%)	0 (0%)
	Localised gingivitis Generalised gingivitis	693 149	570 (82%) 36 (24%)	121 (17%) 88 (59%)	2 (1%) 25 (17%)
***O-R subgroups: iO O-R***	Gingival health	0	0 (0%)	0 (0%)	0 (0%)
	Localised gingivitis Generalised gingivitis	220 35	212 (96%) 13 (37%)	8 (4%) 19 (54%)	0 (0%) 3 (9%)
***Traditional O-R***	Gingival health	487	479 (98%)	8 (2%)	0 (0%)
	Localised gingivitis Generalised gingivitis	473 114	358 (73%) 23 (20%)	113 (24%) 69 (61%)	2 (1%) 22 (19%)
**Manual**	Gingival health	132	132 (100%)	0 (0%)	0 (0%)
	Localised gingivitis Generalised gingivitis	233 82	63 (27%) 4 (4%)	167 (72%) 39 (48%)	3 (1%) 39 (48%)
**Sonic**	Gingival health	344	321 (93%)	23 (7%)	0 (0%)
	Localised gingivitis Generalised gingivitis	457 78	283 (62%) 7 (9%)	166 (36%) 52 (67%)	8 (2%) 19 (24%)

Data shown use 3 classifications: gingival health (<10% bleeding sites), localised gingivitis (10%–30% bleeding sites), and generalised gingivitis (>30% bleeding sites).

O-R, oscillating-rotating.

Figure 1B shows adjusted mean MGI scores at end of treatment for each brush group. A reduction in the average MGI score of 0.29 (95% CI, 0.20–0.37) and 0.16 (95% CI, 0.07–0.24) was observed for all O-R brushes vs manual and sonic brushes, respectively (*P* < .001), representing a 14% and 8% benefit for O-R technology vs the respective controls. When the iO O-R brush subgroup was analysed separately, it demonstrated a comparable MGI score to traditional O-R (3% difference; *P* = .36). The iO O-R and traditional O-R subgroups both showed a statistically significantly lower MGI score vs manual (16% and 13%, respectively) and sonic brushes (8% and 5%, respectively; *P* ≤ .015 for all comparisons). The sonic brush MGI reduction vs manual was 0.13 (*P* = .035), a 6% MGI reduction.

**Plaque:** Network meta-analysis allowed for both direct and indirect comparisons of the treatments with respect to standardised mean plaque score (Table 4). O-R brushes, relative to manual brushes, reduced the average standardised plaque score by 1.85 (95% CI, 1.45–2.26), a reduction of 19%. Relative to sonic brushes, all O-R brushes reduced the average standardised plaque score by 0.57 (95% CI, 0.23–0.92), a reduction of 5%. Sonic brushes, relative to manual brushes, reduced the average standardised average plaque score by 1.28 (95% CI, 0.74–1.81), a reduction of 13%. Standardised plaque scores showed that the iO O-R brush produced the lowest plaque score, followed by the traditional O-R, sonic, and manual brushes (*P* < .001).

**Table 4:** Network meta-analysis on standardised plaque score: standardised mean differences, 95% confidence intervals, and treatment *P* scores.

SMD* (95% confidence interval)	Sonic	All O-R	*P* score
Manual	**−**1.28 (−1.81 to −0.74)	−1.85 (−2.26 to −1.45)	.00
Sonic		−0.57 (−0.92 to −0.23)	.50
All O-R			1.00


SMD* (95% confidence interval)	Sonic	Traditional O-R	iO O-R	*P* score
Manual	**−**1.17 (−1.67 to −0.67)	−1.63 (−2.04 to −1.22)	−2.45 (−3.04 to −1.85)	.00
Sonic		−0.46 (−0.79 to −0.13)	−1.28 (−1.90 to −0.65)	.33
Traditional O-R			−0.82 (−1.45 to −0.19)	.67
iO O-R				1.00

O-R, oscillating-rotating; SMD, standardised mean difference.

⁎ All pairwise comparisons are statistically significant (*P* < .001).

**Regional analysis:** Analyses of lingual, buccal, molar, molar-buccal, and molar-lingual subregions were conducted for number of bleeding sites (Supplementary Figure 2A). Statistically significant differences in favour of all O-R brushes persisted across all analysed subregions (*P* < .001).

Plaque scores were analysed for the same subregions listed above, along with interproximal, interproximal-anterior, interproximal-molar, and gingival surfaces (Supplementary Figure 2B). Statistically significant benefits persisted across all analysed subregions for O-R vs manual. O-R showed statistically significant differences vs sonic for lingual, buccal, interproximal, interproximal-anterior, and interproximal-molar subregions (*P* ≤ .028).

**Fig. 2:**
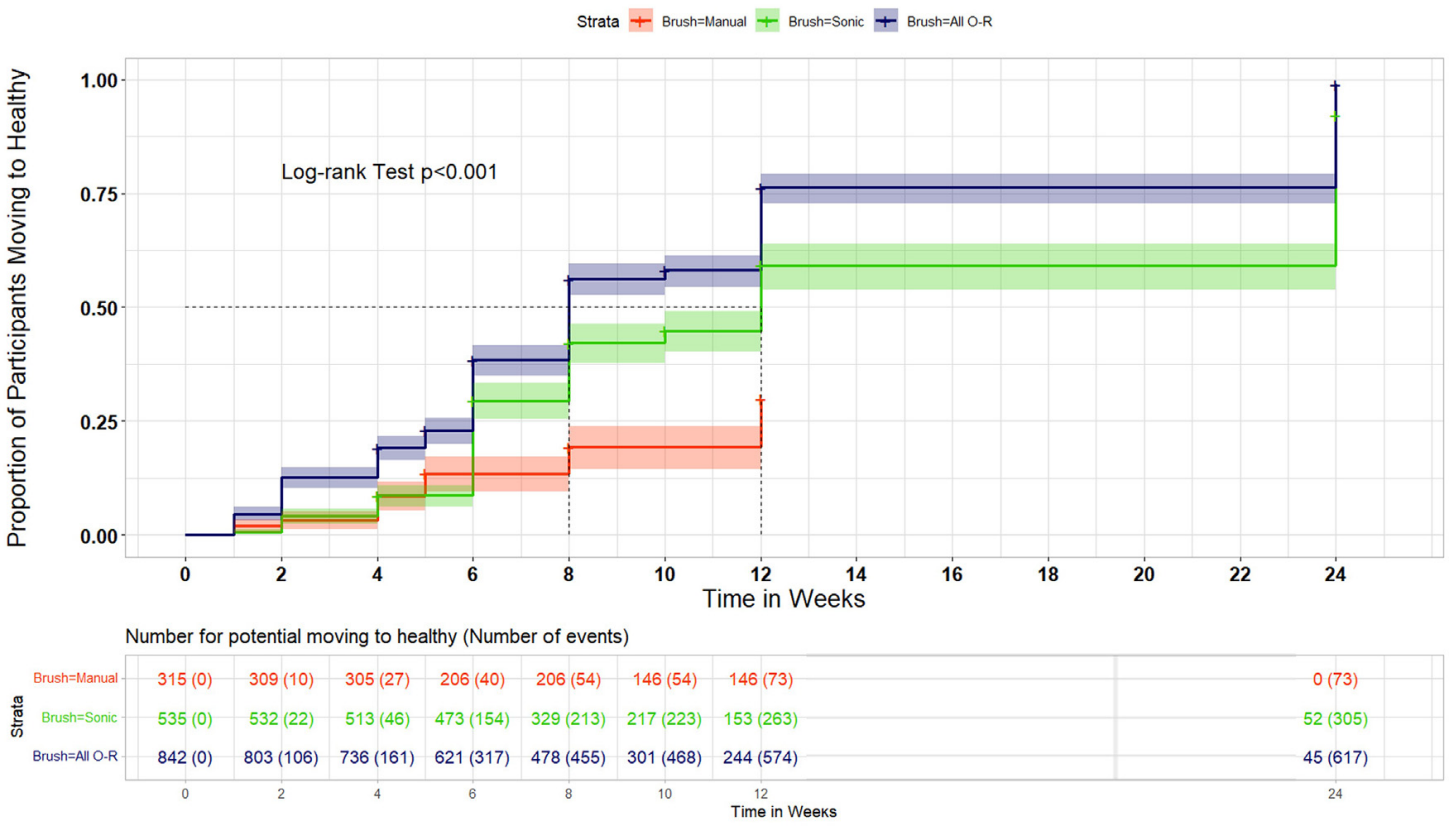
Percentage of participants transitioning to healthy gingival status over time.

**Time to transition to health:** By 12 weeks, a higher percentage of O-R brush users (76.3%; 95% CI, 72.8%−79.4%) than manual brush users (29.8%; 95% CI, 23.9%−35.9%) or sonic brush users (59.2%; 95% CI, 54%−64.1%) transitioned from baseline gingivitis to a state of gingival health (Figure 2). Using the data from all time points, and assessing them cumulatively, the median time to transition to a state of health was 8 weeks for O-R brush users and 12 weeks for sonic brush users.

#### Risk of bias

Risk of bias in each trial was deemed low for all 5 domains (Supplementary Figure 3). Every study was randomised and examiner-blind, and the allocation sequence was unknown during enrollment and treatment assignment, mitigating bias associated with the randomisation process. Each study was also analysed using the per-protocol population to assess the outcome of adhering to intervention, minimising bias associated with deviations from the intended interventions. Bias due to missing outcome data was addressed by availability of data for all, or the vast majority of, participants. Examiners were blinded to treatment and used credentialed clinical measures to assess efficacy, minimising bias in the measurement of the outcome domain. Finally, each study had a prespecified analysis plan that included results irrespective of outcome to mitigate bias in the selection of the reported result.

## Discussion

Results of this meta-analysis confirm consistently greater plaque removal and gingivitis-reduction efficacy of O-R brushes relative to manual and sonic brushes. One factor potentially contributing to efficacy differences is brush head design. O-R brushes have a small, round head, similar to a prophy cup, which contours to the shape of each tooth. Manual toothbrushes typically have a large, rectangular shape, and sonic toothbrushes have preserved a more traditional head shape.

Additionally, for the first time this study demonstrates that the most advanced O-R model provides enhanced bleeding site reduction efficacy relative to traditional O-R brushes. The novel design of the iO O-R brush might contribute to its enhanced effectiveness vs other, “traditional” O-R toothbrushes with respect to transitions to gingival health and bleeding site reduction. The iO O-R toothbrush incorporates a linear magnetic drive system that directs energy to brush filaments tips—eliminating intrinsic losses of energy that are incurred with use of mechanical drive systems in earlier models of oscillating rotating brushes—inducing micro-vibrations at the site of plaque removal.^12^ The superiority of this brush vs manual^28^^,^^29^^,^^50^ and sonic^30^^,^^31^ brushes has already been demonstrated in studies of plaque and gingivitis reduction after as much as 6 months of use; the current results further confirm evidence of the iO brush as an effective oral health tool.

Notably, the relative percentage bleeding site reductions for O-R vs manual and sonic were greater than the respective relative percentage of plaque reduction at the same regions. A given relative reduction in plaque corresponded to a larger relative reduction in the number of bleeding sites. This is consistent with results of previous research suggesting that plaque toxicity may be more important than quantity. The species profile of bacterial plaque, which shows a characteristic, progingivitis shift as early as 24 hours after the cessation of oral hygiene, precedes and correlates with the onset of gingivitis symptoms.^51^ The pathogenic effect of plaque may be independent of total plaque mass due, at least in part, to lipopolysaccharides that are present on the uniquely problematic subsets of plaque bacteria.^52^

The results of this meta-analysis are consistent with those of multiple other systematic reviews and meta-analyses. Whilst some groups have reported little to no efficacy difference between O-R and sonic brushes, including high-frequency models, with respect to plaque or gingivitis reduction,^53^^,^^54^ a large body of independently conducted research supports the superior performance of O-R brushes vs sonic as well as manual brushes.^8^, ^9^, ^10^^,^^14^^,^^16^^,^^17^ The consistently lower plaque effects seen for manual toothbrushes vs electric toothbrushes illustrate that whilst a skilled user can achieve thorough plaque removal with a manual toothbrush, it is difficult to do in practice.^7^

A limitation of the RCTs included in this meta-analysis is the lack of double-blinding due to logistical challenges associated with concealing toothbrush identity. However, all trials were examiner-blinded. Another limitation is that every trial did not have a positive and negative control toothbrush. The fact that all trials were supported by a single manufacturer and the meta-analysis was not conducted in conjunction with a systematic review might be considered a potential limitation. However, access to participant-level data for each study not only was necessary to complete the set of analyses but also provided the advantage of producing distinctive assessments such as the transition-to-health segmentations. Risk of bias was deemed low for all studies using the Cochrane RoB2 assessment tool.^19^ All studies were randomised, controlled, and examiner-blinded and used credentialed research standards. The majority of studies were conducted at independent sites according to ICH/GCP standards. Findings for 22 of 26 total studies have been published in peer-reviewed journals,^28^, ^29^, ^30^, ^31^, ^32^, ^33^, ^34^, ^35^, ^36^, ^37^, ^38^, ^39^, ^40^, ^41^, ^42^, ^43^, ^44^, ^45^, ^46^, ^47^, ^48^, ^49^ mitigating across-study risk of bias. All studies were analysed per a prespecified analysis plan using the per-protocol population to evaluate the effect of complying with intervention. The scale and rigour of the dataset supports its validity and reproducibility, and the clinical methods are validated. A core strength of these analyses is the large and diverse population, making the findings applicable to the broader global population.

The O-R brush examined in the current meta-analysis features an interactive app. The few studies to date that explored such apps suggest that they lead to brushing behaviour (eg, brushing coverage, time, pressure) and oral health improvements.^55^, ^56^, ^57^ Future research could further explore behaviour and health benefits of toothbrush apps. Another area for further investigation is regimen research. Whilst O-R electric brush technology provides significant advances in plaque removal, antibacterial dentifrice and rinse can inhibit plaque regrowth between brushing sessions to enhance plaque control.^58^^,^^59^

In conclusion, this meta-analysis shows that O-R toothbrushes provide significantly better plaque and gingivitis reduction and a faster transition to health than sonic or manual brushes. Relative to other O-R brushes, the most advanced O-R model produces a significantly greater rate of transition to gingival health and significantly greater reductions in number of bleeding sites and plaque scores.

## Author contributions

All authors have made substantial contributions to the conception and design of the study. YZ and JG led data collection and analysis. All authors were involved in data interpretation, drafting the manuscript and revising it critically and have given final approval of the version to be published.

## Funding

Procter & Gamble funded the analysis and medical writing assistance.

## Conflict of interest

Drs Zou and Grender are employees of The Procter & Gamble Company. Dr Adam is an employee of Procter & Gamble Service GmbH. Dr Levin has done consulting work for The Procter & Gamble Company.

## Appendix Supplementary materials

Image, application 1

## Footnotes

Supplementary material associated with this article can be found in the online version at doi:10.1016/j.identj.2023.06.009.
