# Supplementary material for: A Meta-analysis Comparing Toothbrush Technologies on Gingivitis and Plaque
Source: Int Dent J. 2023 Jul 21;74(1):146–56. doi: 10.1016/j.identj.2023.06.009 (PMC10829363; doi:10.1016/j.identj.2023.06.009)

Supplementary Figure 1. Gingival bleeding response versus baseline bleeding levels: Traditional O-R and iO O-R versus manual and sonic brushes. End-of-treatment bleeding site differences between toothbrush types are consistent across the wide range of baseline bleeding levels.

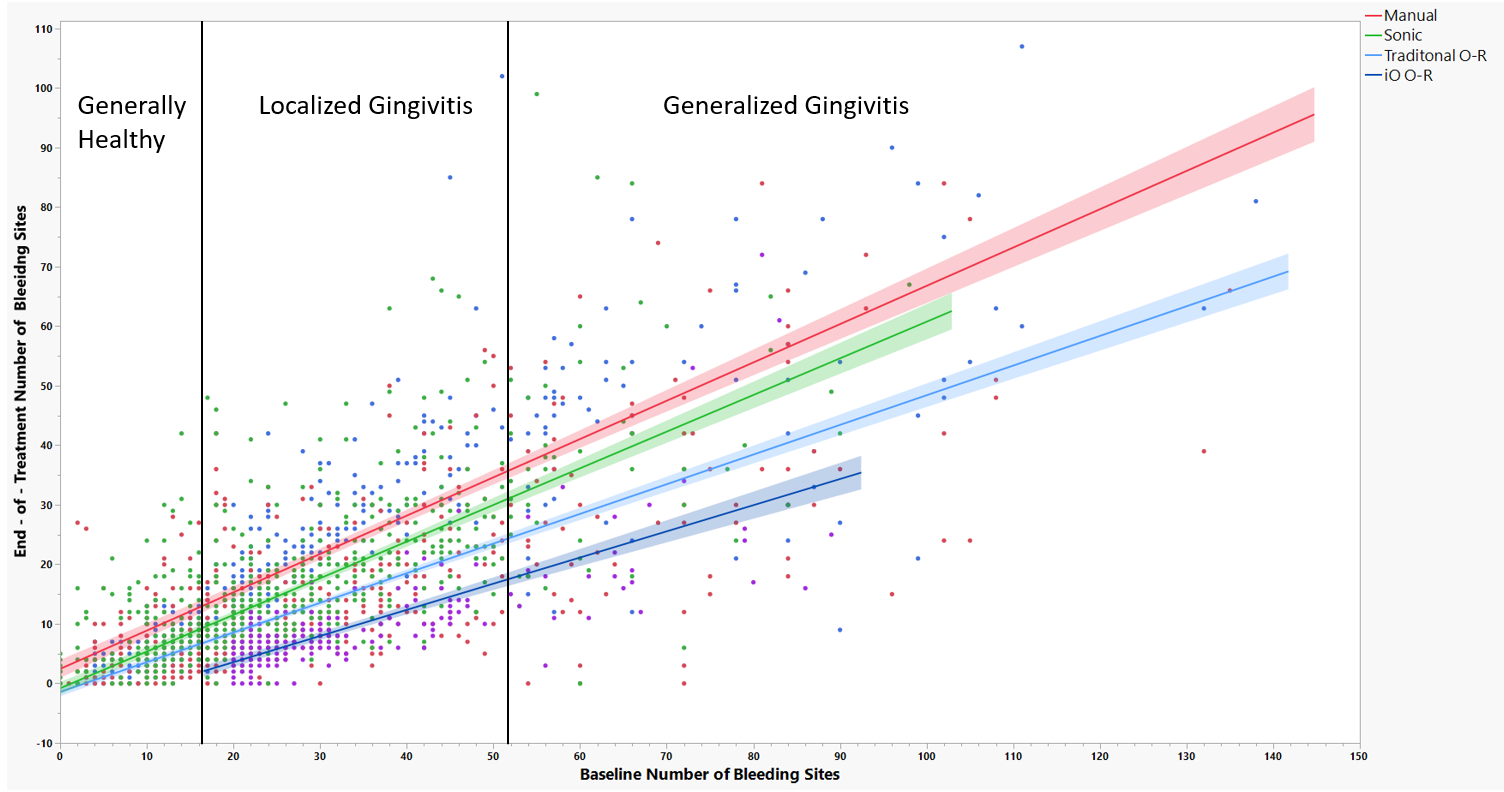


iO O-R brush had smallest # of EOT bleeding sites across all baseline bleeding levels

Manual had highest # of EOT bleeding sites across all baseline bleeding levels

Supplementary Figure 2a. Percent change in adjusted mean number of bleeding sites for O-R versus manual and sonic controls, analyzed by subregion. Differences between treatments were statistically significantly different (*P* < .001), favoring the O-R brush, for all data shown.

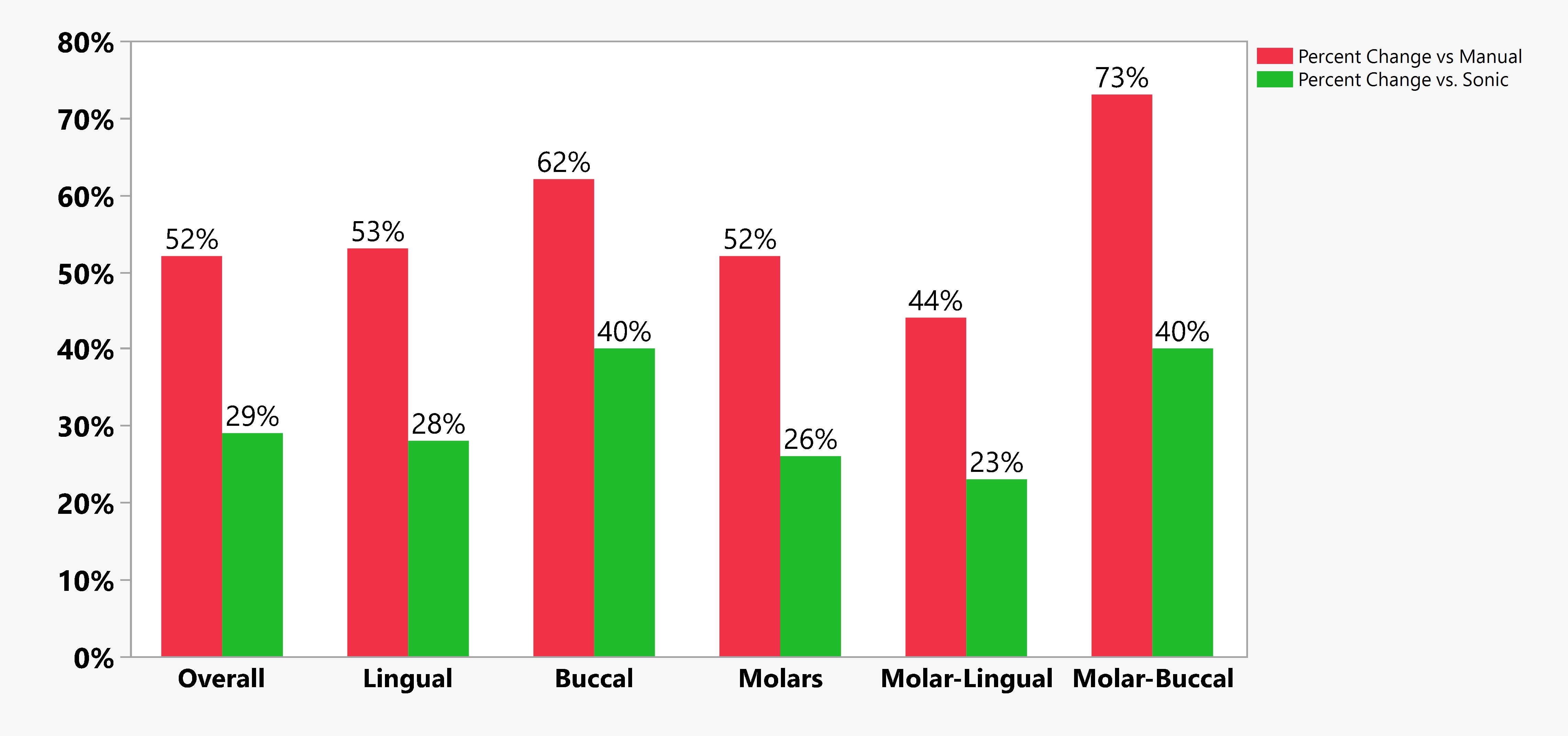


Supplementary Figure 2b. Percent change in adjusted mean RMNPI score for O-R versus manual and sonic controls, analyzed by subregion. Differences between treatments were statistically significantly different (*P* < .028), favoring the O-R brush, for all data shown. If the difference between treatments is not shown in the figure, it was not statistically significant (*P* > .05) in that region.

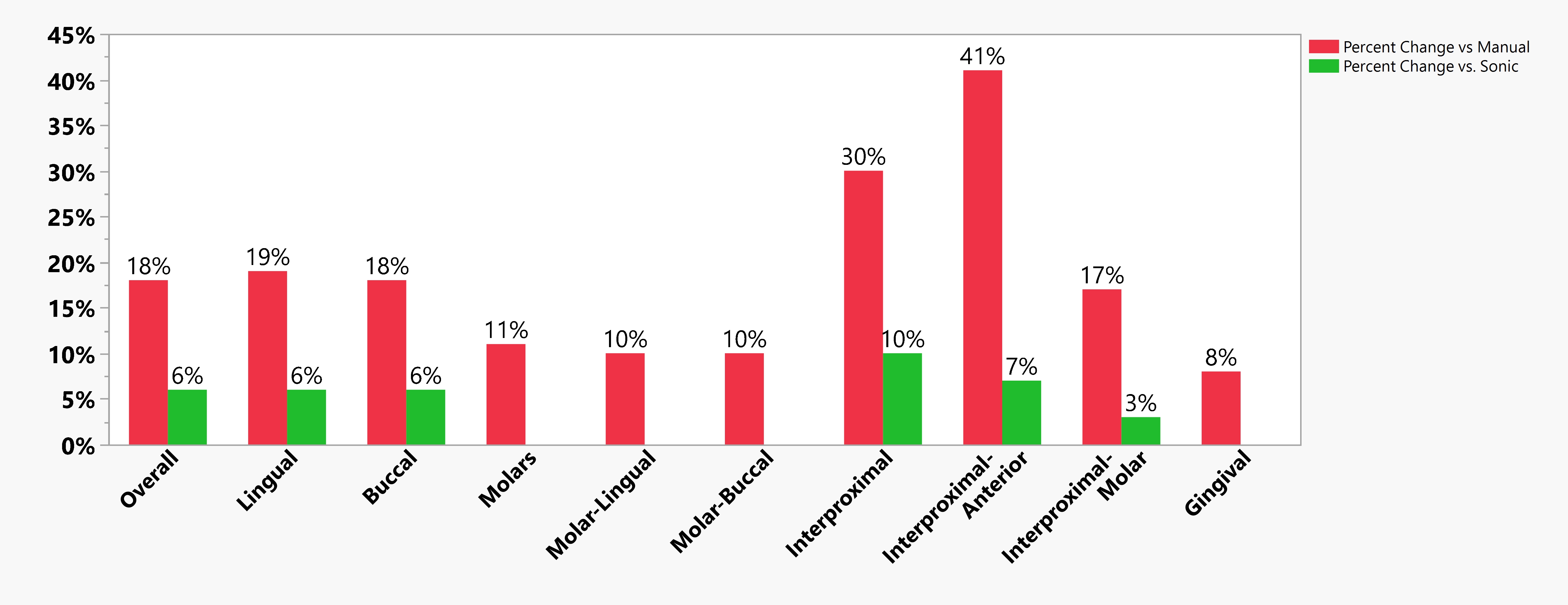


Supplementary Figure 3. Risk of Bias Results


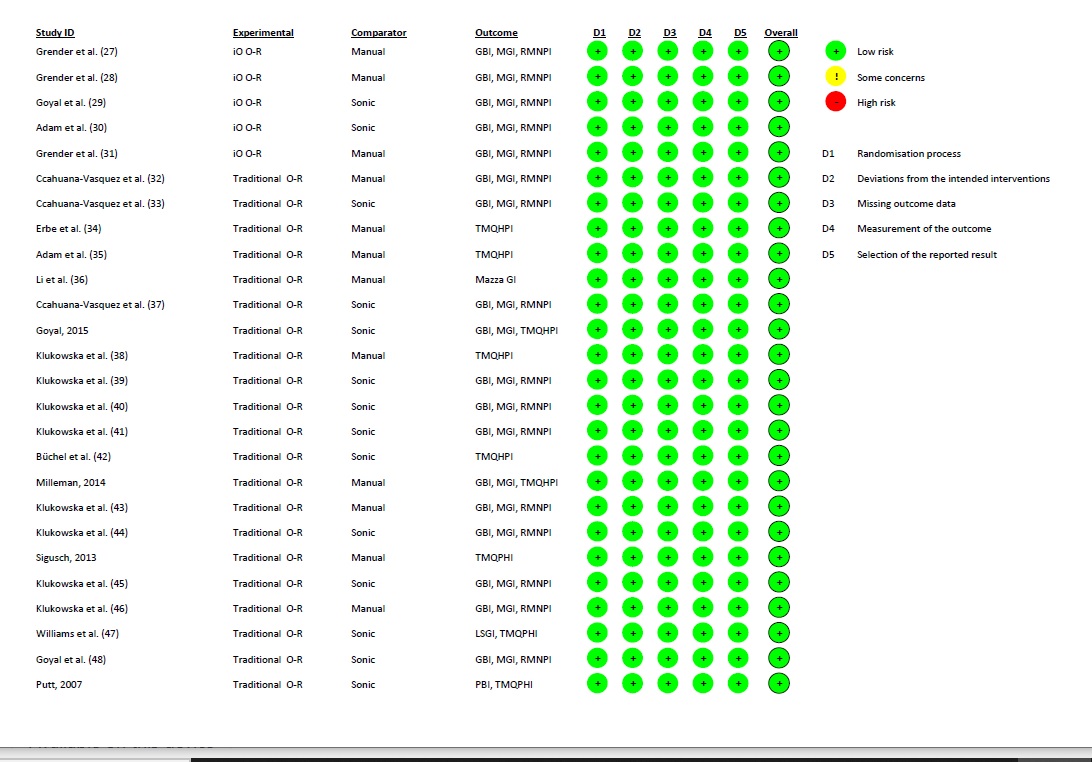

Supplement: Supplementary file 1 [file mmc1.docx]
